# Biases in the Simulation and Analysis of Fractal Processes

**DOI:** 10.1155/2019/4025305

**Published:** 2019-12-03

**Authors:** Clément Roume, Samar Ezzina, Hubert Blain, Didier Delignières

**Affiliations:** ^1^Euromov, University Montpellier, 700 Avenue du Pic Saint Loup, 34090 Montpellier, France; ^2^Union Nationale Sportive Léo Lagrange, 150 rue des Poissonniers, 75883 Paris, France; ^3^Department of Internal Medicine and Geriatrics, Montpellier University Hospital, 39 Avenue Charles Flahault, 34090 Montpellier, France

## Abstract

Fractal processes have recently received a growing interest, especially in the domain of rehabilitation. More precisely, the evolution of fractality with aging and disease, suggesting a loss of complexity, has inspired a number of studies that tried, for example, to entrain patients with fractal rhythms. This kind of study requires relevant methods for generating fractal signals and for assessing the fractality of the series produced by participants. In the present work, we engaged a cross validation of three methods of generation and three methods of analysis. We generated exact fractal series with the Davies–Harte (DH) algorithm, the spectral synthesis method (SSM), and the ARFIMA simulation method. The series were analyzed by detrended fluctuation analysis (DFA), power spectral density (PSD) method, and ARFIMA modeling. Results show that some methods of generation present systematic biases: DH presented a strong bias toward white noise in fBm series close to the 1/*f* boundary and SSM produced series with a larger variability around the expected exponent, as compared with other methods. In contrast, ARFIMA simulations provided quite accurate series, without major bias. Concerning the methods of analysis, DFA tended to systematically underestimate fBm series. In contrast, PSD yielded overestimates for fBm series. With DFA, the variability of estimates tended to increase for fGn series as they approached the 1/*f* boundary and reached unacceptable levels for fBm series. The highest levels of variability were produced by PSD. Finally, ARFIMA methods generated the best series and provided the most accurate and less variable estimates.

## 1. Introduction

The repeated measurement of physiological or behavioral events (stride durations, heartbeat intervals, and intertap intervals) is typically characterized by a marked variability. For a long time, this variability has just been considered a random perturbation, without any functional signification. However, a number of authors, during the last two decades, showed that these biological series presented a typical long-range correlational structure over time and especially a statistical self-similar (fractal) pattern [1–4]. Fractal processes have recently received a growing interest, especially in the domain of rehabilitation. More precisely, the evolution of fractality with aging and disease, suggesting a loss of complexity [5], has inspired a number of studies that tried, for example, to entrain patients with fractal rhythms [6–8].

In this domain, authors are confronted with two main methodological problems: The first one concerns the evaluation of the level of long-range correlations in physiological series. A number of different methods have been proposed, and their respective qualities were systematically assessed in comparative studies [9–12]. The second one refers to the generation of exact fractal signals necessary of providing experimental devices (metronomes and virtual environments) with controlled long-range correlation properties. A number of methods have been proposed for simulating such series [13–15]. The assessment of estimation methods, on the one hand, and simulation methods, on the other hand, raises a typical problem of circularity, performances, and biases in the former being analyzed on the basis of the latter, and vice versa [11]. When a bias is identified, it remains difficult to attribute the problem to the method of simulation or to the method of assessment. In order to overcome this problem, we propose in the present paper a cross-validation study, combining three methods of generation and three methods of analysis.

We first propose a formal introduction of the three main domains of definition of long-range correlated processes: the fractional Brownian motion framework [16], the spectral domain [17], and the autoregressive fractionally integrated moving average (ARFIMA) processes [18].

## 2. Theoretical Models

### 2.1. The fBm/fGn Model

The *fractional Brownian motion* (fBm) denoted as *B*_*H*_(*t*) is a mathematical model of continuous stochastic process introduced by Mandelbrot and Van Ness [16] as a generalization of the Brownian motion, where increments do not need to be independent. *B*_*H*_(*t*) is characterized by the Hurst parameter (*H*), which can take any real value within the interval ]0, 1[. This value gives information about the nature and the strength of the correlation between successive increments in the process. If *H* is below 0.5, *B*_*H*_(*t*) is underdiffusive, and its increments are anticorrelated. In contrast, if *H* is above 0.5, *B*_*H*_(*t*) is overdiffusive, and its increments are positively correlated. In the case that *H* equals 0.5, *B*_0.5_(*t*) is an ordinary Brownian motion (normal diffusion), and its increments are an uncorrelated Gaussian white noise.

fBm has some fundamental properties. The first one is that its variance grows as a power function of the length of the time interval observed, with an exponent 2*H*:

(1) $$\begin{array}{c} V_{B_{H}}\propto \Delta t^{2H}. \end{array}$$

The second is that fBm is a fractal process, characterized by statistical self-similarity:

(2) $$\begin{array}{c} B_{H}\left(at\right)\equiv a^{H}B_{H}\left(t\right),\;\forall a\text{ and }t>0. \end{array}$$

By definition, *B*_*H*_(*t*) is fully described by its autocovariance function *γ*_*B*_*H*__(*t*, *s*):

(3) $$\begin{array}{c} \gamma_{B_{H}}\left(t,s\right)=\mathrm{cov}\left(B_{H}\left(t\right),B_{H}\left(s\right)\right)=\frac{V_{B_{H}}}{2}\left({\left|t\right|}^{2H}\;+\;{\left|s\right|}^{2H}\;-\;{\left|t-s\right|}^{2H}\right). \end{array}$$

As previously indicated, the successive increments of *B*_*H*_(*t*) can be correlated and the Hurst exponent informs about the nature of this correlation, and thus, the derivative of *B*_*H*_(*t*) should be a stationary correlated noise. However, this derivative cannot be calculated because in theory Brownian motion describes an infinitely broken continuous trajectory. In other words, nondifferentiability is a fundamental property of *B*_*H*_(*t*). One can still estimate this derivative using a discrete version of *B*_*H*_(*t*) where increments are defined on a time interval *m*. This estimate is a discrete process called *fractional Gaussian noise* (fGn), denoted as *G*_*m*_(*i*):

(4) $$\begin{array}{c} G_{m}\left(i\right)=B_{H}\left(t\right)-B_{H}\left(t-m\right). \end{array}$$

As for the fBm, the fGn is fully described by its autocovariance function, which is easily derived from equation (3) with *m* = 1:

(5) $$\begin{array}{c} \gamma_{G_{1}}\left(\tau \right)=\mathrm{cov}\left(G_{1}\left(i\right);G_{1}\left(i+\tau \right)\right)=\frac{V_{G_{1}}}{2}\left({\left|\tau +1\right|}^{2H}-2{\left|\tau \right|}^{2H}\;+\;{\left|\tau -1\right|}^{2H}\right). \end{array}$$

### 2.2. The Spectral Model

Stochastic fractal processes can also be defined in the frequency domain, on the basis of a scaling law that relates power (i.e., squared amplitude) to frequency according to an inverse power function, with an exponent *β* [9, 10]. For an fGn process with exponent *H*, the power spectrum can be expressed as follows [12, 19]:

(6) $$\begin{array}{c} S\left(f\right)\propto \frac{1}{f^{2H-1}}, \end{array}$$

and for the corresponding fBm process [12, 20]:

(7) $$\begin{array}{c} S\left(f\right)\propto \frac{1}{f^{2H+1}}. \end{array}$$

Then the power spectrum of fGn/fBm processes has the general form:

(8) $$\begin{array}{c} S\left(f\right)\propto \frac{1}{f^{\beta }}, \end{array}$$

with *β* ∈ ]−1, 1[ for fGn and *β* ∈ ]1, 3[ for fBm. This suggests that fGn and fBm could be considered as a continuum, with both families being characterized in the time domain by a scaling exponent *α*, *α* = *H* for fGn and *α* = *H* + 1 for fBm. This assumption has been exploited by Peng et al. [4] in the conception of the detrended fluctuation analysis (see Section 3). The scaling exponent *α* is linearly related to the spectral exponent *β* over the whole fGn/fBm continuum by *α*=(*β* + 1/2) [21]. The case *β* = 1 defines the so-called “1/*f* noise,” which represents the boundary between fGn and fBm processes.

### 2.3. The ARFIMA Model

A third approach to long-range correlated processes is provided by the autoregressive fractionally integrated moving average (ARFIMA) models [18]. This approach is an extension of the ARIMA (for autoregressive, integrated, moving average) framework, introduced by Box et al. [22], which intended to represent a variety of short-term relationships in time series. ARIMA models are potentially composed of three components. The autoregressive component suggests that the current observation *y*(*t*) is determined by a weighted sum of the *p* previous observations, plus a random perturbation *ε*:

(9) $$\begin{array}{c} y\left(t\right)=\overset{p}{\underset{k=1}{\sum }}\phi \left(k\right)y\left(t-k\right)+\varepsilon \left(t\right). \end{array}$$

The moving average component supposes that the current observation depends on the value of the random perturbations that affect the *q* preceding observations, plus its own specific perturbation:

(10) $$\begin{array}{c} y\left(t\right)=\overset{q}{\underset{k=1}{\sum }}\theta \left(k\right)\varepsilon \left(t-k\right)+\varepsilon \left(t\right). \end{array}$$

Finally, the differencing parameter *d* indicates the number of differencing that should be applied to the series before modeling. An ARIMA model is the combination of these three components and can be designated by the respective orders of the three processes as (*p*, *d*, *q*). ARIMA modeling has been used either for generating time series with specified *p*, *d*, *q* parameters or for determining the best *p*, *d*, *q* combination for accounting for a given series [22, 23].

ARIMA models could be more conveniently expressed using the so-called *backshift operator*, defined as

(11) $$\begin{array}{c} Bx\left(t\right)=x\left(t-1\right). \end{array}$$

The generic ARIMA (*p*, *d*, *q*) model can then be rewritten as

(12) $$\begin{array}{c} \phi \left(B\right){\left(1-B\right)}^{d}x\left(t\right)=\theta \left(B\right)\varepsilon \left(t\right), \end{array}$$

where *ϕ*(*B*) and *θ*(*B*) are, respectively, the autoregressive and the moving average operators, represented as polynomials in the backshift operator: *ϕ*(*B*)=1 − *Bϕ*(1) − *B*^2^*ϕ*(2) − ⋯− *B*^*p*^*ϕ*(*p*) and *θ*(*B*)=1+*Bθ*(1)+ *B*^2^*θ*(2)+⋯+*B*^*q*^*θ*(*q*) [24]. In the initial formulation of the model, the *d* parameter was an integer [22]. Granger and Joyeux [18] showed that it was possible to provide this model with long-range dependence properties by allowing the differencing parameter *d* to take on fractional values, thereby obtaining an ARFIMA model.

Here we focus on the most simple model ARFIMA(0, *d*, 0), which is supposed to only contain long-range correlations. Using the backshift operator notation, this model is expressed as follows [25]:

(13) $$\begin{array}{c} {\left(1-B\right)}^{d}x\left(t\right)=\varepsilon \left(t\right), \end{array}$$

with

(14) $$\begin{array}{c} {\left(1-B\right)}^{d}=\overset{\infty }{\underset{k=0}{\sum }}\left(\begin{array}{c} d\\ k \end{array}\right){\left(-1\right)}^{k}B^{k}. \end{array}$$

Granger and Joyeux [18] derive a filter *A*(*B*) from equations (13) and (14) and demonstrate that the process can be rewritten as

(15) $$\begin{array}{c} x\left(t\right)=A\left(B\right)\varepsilon \left(t\right)=\overset{\infty }{\underset{k=0}{\sum }}a\left(k\right)\varepsilon \left(t-k\right), \end{array}$$

(16) $$\begin{array}{c} a\left(k\right)=\frac{\Gamma \left(k+d\right)}{\Gamma \left(k+1\right)\Gamma \left(d\right)}. \end{array}$$

where *d* is a measure of the intensity of long-range correlations in the series. Note, however, that ARFIMA models account only for stationary series, *d* being bounded within the interval ]−0.5; 0.5[. In other words, ARFIMA models remain limited to fGn series. *d* is related to the spectral exponent *β* and the scaling exponent *α* by the following linear equations:

(17) $$\begin{array}{c} \beta =2d,\\ \alpha =\left(\frac{2d+1}{2}\right). \end{array}$$

Each of these theoretical frameworks provided specific methods for generating fractal signals or for assessing the fractality of empirical series. In the present work, we engaged a cross validation of three methods of generation and three methods of analysis. We selected one simulation method and one estimation method in each previously presented domain of definition. Our rational is that biases that are revealed by the three analysis methods should be attributed to the generation method and conversely biases that appear whatever the generation method should originate in the analysis method.

## 3. Methods

In order to explore the whole fGn/fBm continuum, we first generated series from *α* = 0.1 to 0.9, by steps of 0.1, and from 1.01 to 1.9, by steps of 0.1. These values were used in most previous similar studies [9, 10, 26]. Additionally, in order to analyze more closely the behavior of simulation and analysis methods around to the 1/*f* boundary, we generated a series from *α* = 0.91 to 1.09, by steps of 0.01. This range of exponents was rarely considered in the literature (for a noticeable exception, see [27]). However, this focus on the 1/*f* boundary seems of particular interest, because the problems of fGn/fBm classification are concentrated within this interval [10] and also because one could have some doubts about the hypothesis of continuity around the 1/*f* boundary [28].

We used three methods of generation: the Davies–Harte algorithm, the spectral synthesis method, and the ARFIMA simulation method. These methods are detailed below. For each selected *α* value and with each method, we generated 120 series of 1024 data points. In this section, all methods are written in the discrete time and frequency domain; for reading convenience, we keep the variable *t* for discrete time domain and *f* for discrete frequency domain.

### 3.1. Davies–Harte Algorithm (DH)

We used the algorithm proposed by Davies and Harte [13], for generating fGn series of length *N* (*N* being a power of 2). As previously indicated, an fGn process is fully described by its autocovariance function (see equation (5)). Then, one can deduce the exact spectral power *S* expected for this autocovariance function, from the discrete Fourier transform of the following sequences of covariance values *γ*_*G*1_ defined by equation (5): *γ*_*G*1_(0), *γ*_*G*1_(1),…, *γ*_*G*1_((*N*/2)−1); *γ*_*G*1_(*N*/2), *γ*_*G*1_((*N*/2)−1),…, *γ*_*G*1_(1):

(18) $$\begin{array}{c} S\left(f\right)=\overset{\left(N/2\right)-1}{\underset{\tau =0}{\sum }}\gamma_{G_{1}}\left(\tau \right)e^{-i2\pi f\left(\tau /N\right)}+\overset{N-1}{\underset{\tau =N/2}{\sum }}\gamma_{G_{1}}\left(N-\tau \right)e^{-i2\pi f\left(\tau /N\right)}, \end{array}$$

where *f* = 0, 1,…, *N* − 1. It is important to check that *S*(*f*) ≥ 0 for all *f*. Negativity would indicate that the sequence is not valid.

Let *W*_gn_(*f*), where *f* = 0,…, *N* − 1, be a white Gaussian noise. The randomized spectral amplitudes, *V*(*f*), are calculated according to the following equations:

(19) $$\begin{array}{c} V\left(0\right)=\sqrt{\text{NS}\left(0\right)}\times W_{\text{gn}}\left(0\right),\\ V\left(f\right)=\sqrt{\frac{\text{NS}\left(f\right)}{2}}\times \left(W_{\text{gn}}\left(2f-1\right)+iW_{\text{gn}}\left(2f\right)\right)\text{ for }1\leq f\leq \frac{N}{2},\\ V\left(\frac{N}{2}\right)=\sqrt{\text{NS}\left(\frac{N}{2}\right)}\times W_{\text{gn}}\left(N-1\right),\\ V\left(f\right)=\sqrt{\frac{\text{NS}\left(f\right)}{2}}\times \left(W_{\text{gn}}\left(2N-1-2f\right)-iW_{\text{gn}}\left(2N-2f\right)\right)\;\text{ for }\frac{N}{2}+1\leq f\leq N-1. \end{array}$$

Finally, the first *N* elements of the discrete Fourier transform of *V* are used to compute the simulated series *x*(*t*):

(20) $$\begin{array}{c} x\left(t\right)={ℑ}^{-1}\left\{V\left(f\right)\right\}=\frac{1}{N}\overset{N-1}{\underset{f=0}{\sum }}V\left(f\right)e^{i2\pi f\left(t/N\right)}, \end{array}$$

where *t* = 1, 2,…, *N*.

We first generated fGn series for *H* values ranging from 0.1 to 0.9, by steps of 0.1. In order to explore more precisely the performance of DFA close to the 1/*f* boundary, we also generated fGn series for *H* values ranging from 0.91 to 0.99, by steps of 0.01. A second set of fGn series was generated, for *H* values ranging from 0.1 to 0.9, by steps of 0.1, and was integrated for obtaining fBm series for each corresponding *H* value (i.e., for *α* values ranging from 1.1 to 1.9). Finally, we generated fGn series for *H* ranging from 0.01 to 0.09 and integrated them for obtaining fBm series close to the 1/*f* boundary (i.e., for *α* values ranging from 1.01 to 1.09).

### 3.2. Spectral Synthesis Method (SSM)

The spectral synthesis method is designed to produce fBm and fGn series *x*(*t*) based on the characteristics of the power spectral density *S*(*f*) of these signals. In other words, the idea of SSM is to generate a right kind of *S*(*f*) that gives rise to fBm or fGn with an exponent 0 *<* *H* *<* 1.

Since *S*(*f*) is obtained as the square of the modulus of the Fourier transform of the signal and its proportional to *f*^−*β*^ (see equation (8)),

(21) $$\begin{array}{c} S\left(f\right)={\left|X\left(f\right)\right|}^{2}\propto f^{-\beta }. \end{array}$$

We need to generate a complex series *X*(*f*) with a modulus *r*(*f*) proportional to 1/*f*^*β*/2^. *r*(*f*) is obtained as follows:

(22) $$\begin{array}{c} r\left(f\right)=\frac{W_{\text{gn}}\left(f\right)}{f^{\beta /2}}, \end{array}$$

where *W*_gn_ is a white Gaussian noise and *f* is the frequency ranging from 1 to *N*/2.

To generate the *X*(*f*), we also need to generate a random phase in radian:

(23) $$\begin{array}{c} \phi \left(f\right)=2\pi W_{\text{un}}, \end{array}$$

where *W*_un_ is a white noise with uniform distribution.

We can create the complex coefficients:

(24) $$\begin{array}{c} a\left(f\right)=r\left(f\right)\cos\;\phi \left(f\right),\\ b\left(f\right)=r\left(f\right)\sin\;\phi \left(f\right). \end{array}$$

These coefficients are extended to the whole range of expected values (in respect of the Shannon theorem), *a*(*f*) from (*N*/2)+ 1 to *N* being equal to *a*(*f*) from (*N*/2) to 1 and *b*(*f*) from (*N*/2)+ 1 to *N* being equal to *b*(*f*) from (*N*/2)to 1. Then, the complex series *X*(*f*) are generated as follows:

(25) $$\begin{array}{c} X\left(f\right)=a\left(f\right)+ib\left(f\right). \end{array}$$

Finally, the inverse Fourier transform of this complex number is computed to obtain the time series:

(26) $$\begin{array}{c} x\left(t\right)={ℱ}^{-1}\left\{X\left(f\right)\right\}=\frac{1}{N}\overset{N}{\underset{f=1}{\sum }}X\left(f\right)e^{i2\pi f\left(t/N\right)}, \end{array}$$

where *t* = 1, 2,…, *N*.

### 3.3. ARFIMA Simulation Method

We used the Matlab code proposed by Fatichi [14], based on equation (12), and in the present case, as we limited ourselves to ARFIMA (0, *d*, 0) models, on equation (13). This code just bounds the summation in equation (15) to *k* = 100. We used this procedure for generating fGn series (*d* ∈ ]−0.5, 0.5[). In order to obtain the whole set of series we needed, we computed fBm series by cumulated summation.

We now present the three estimation methods we used. Note that for a better readability, the exponents of the simulated series and the estimates are expressed or converted in *α* metrics. Estimates are denoted with a circumflex ($\hat{\alpha }$, $\hat{\beta }$, $\hat{d}$).

### 3.4. Detrended Fluctuation Analysis

The DFA algorithm works as follows, for a series *x*(*t*) of length *N* where *t* = 1, 2,…, *N*. The series is first integrated, by computing for each *t* the accumulated departure from the mean of the whole series:

(27) $$\begin{array}{c} X\left(t\right)=\overset{t}{\underset{i=1}{\sum }}\left(x\left(i\right)-\bar{x}\right). \end{array}$$

This integrated series is then divided into *k* nonoverlapping intervals of length *n*. The last *N* *−* *kn* data points are excluded from analysis. Within each interval, a least squares line is fitted to the data. The series *X*(*t*) is then locally detrended by subtracting the theoretical values *X*^th^(*t*) given by the regression. For a given interval length *n*, the characteristic size of fluctuation for this integrated and detrended series is calculated by the following equation:

(28) $$\begin{array}{c} F\left(n\right)=\sqrt{\frac{1}{N-kn}\overset{N-kn}{\underset{t=1}{\sum }}{\left(X\left(t\right)-X^{\text{Th}}\left(t\right)\right)}^{2}}. \end{array}$$

In the original algorithm, this computation is repeated over all possible interval lengths, for example, from *n* = 10 to *n* = *N*/2 [9]. In the present paper, we applied the *evenly spaced averaged* version of DFA [29], which significantly reduces the variability of estimates. This procedure consists in dividing the (log) abscissa into *P* bins of length (log_10_(*n*_max_/*n*_min_))/*P*, starting from log_10_(*n*_min_). The *P* bins are defined as follows:

(29) $$\begin{array}{c} \text{bin}\left(p\right)=\left[\log_{10}\left(n_{\min}\right)+\left(\frac{p-1}{P}\right)\left(\log_{10}\left(\frac{n_{\max}}{n_{\min}}\right)\right);\log_{10}\left(n_{\min}\right)+\frac{p}{P}\left(\log_{10}\left(\frac{n_{\max}}{n_{\min}}\right)\right)\right], \end{array}$$

where *p* = 1, 2,…, *P*. In the present analyses, we set *n*_min_ = 10, *n*_max_ = 512, and *P* = 18.

Within each bin *p*, the average interval length $\bar{n}\left(p\right)$ and the average fluctuation size $\bar{F}\left(n\left(p\right)\right)$ are computed. A power law is expected as

(30) $$\begin{array}{c} \bar{F}\left(n\left(p\right)\right)\propto {\left(\bar{n}\left(p\right)\right)}^{\alpha }. \end{array}$$

The exponent estimate ($\hat{\alpha }$) is obtained as the slope of the double logarithmic plot of $\bar{F}\left(n\left(p\right)\right)$ as a function of $\bar{n}\left(p\right)$.

### 3.5. Power Spectral Density Analysis (PSD)

This method works on the basis of the periodogram obtained by the fast Fourier transform algorithm and exploits the power law given by equation (8). $\hat{\beta }$ is estimated by calculating the negative slope (−*β*) of the line relating log (*S*(*f*)) to log *f*.

In the present paper, we used the improved version proposed by Fougere [30] and modified by Eke et al. [10], designated as ^low^PSD_we_. This method uses a combination of preprocessing operations: first, the mean of the series is subtracted from each value, and then, a parabolic window is applied—each value in the series is multiplied by the following function:

(31) $$\begin{array}{c} \left[\begin{array}{c} W\left(t\right)=1-{\left(\frac{2t}{N\;+\;1}-1\right)}^{2},\\ x_{w}\left(t\right)=x\left(t\right)\times W\left(t\right), \end{array}\right. \end{array}$$

where *t* = 1, 2,…, *N*.

Secondly, a bridge detrending is performed by subtracting from the data the line connecting the first and last point of the series:

(32) $$\begin{array}{c} \left[\begin{array}{c} l\left(t\right)=\frac{\left(x_{w}\left(1\right)-x_{w}\left(N\right)\right)\times \left(t-1\right)}{N-1}+x_{w}\left(N\right),\\ x_{w,l}\left(t\right)=x_{w}\left(t\right)-l\left(t\right), \end{array}\right. \end{array}$$

where *t* = 1, 2,…, *N*.

The Fourier transform is applied on the modified series *x*_*w*,*l*_, and the fitting of *β* excludes the high-frequency power estimates (*f* > 1/8 of maximal frequency). This method was proven by Eke et al. [10] to provide more reliable estimates of the spectral index. For allowing a direct comparison between methods, *β* was then converted into *α* metrics by a simple linear transform $\left(\hat{\alpha }=\left(\hat{\beta }+1\right)/2\right)$.

### 3.6. ARFIMA Modeling

The *d* parameter was estimated by fitting an ARFIMA(0, *d*, 0) model to the series using Whittle approximation of the maximum likelihood estimator. We used the ARFIMA(*p*, *d*, *q*) estimator package for Matlab proposed by Inzelt [31] on the Matlab central file exchange platform.

As previously explained, ARFIMA modeling holds only for fGn series and *d* is bounded within the interval ]−0.5, 0.5[. For fBm series, Diebolt and Guiraud [32] proposed to apply ARFIMA modeling to the corresponding fGn (obtained by differentiation) and then to estimate the theoretical fractional parameter of the fBm series by adding 1 to the *d* value obtained from the fGn. This strategy, however, requires an a priori assessment of the fGn/fBm classification of series. Some solutions, based on the preliminary application of methods working indifferently on fGn and fBm, such as DFA or PSD, have been proposed but yielded important percentages of misclassification around the 1/*f* boundary [9, 10].

In order to apply ARFIMA modeling indifferently on fGn and fBm series, as DFA and PSD, we used the following strategy: the algorithm consists in finding the best Whittle approximate of the maximum likelihood estimator by constrained optimization. According to ARFIMA properties, the output parameter is bounded, yielding the parameter value $\hat{d}=0.4999$ if the algorithm did not converge on the upper bound. When ${\hat{d}}_{\text{o}}$, obtained from the original series, was equal to 0.4999, the series was considered as fBm and the algorithm was applied on the differentiated time series in order to obtain ${\hat{d}}_{\text{diff}}$ estimate. In this nonstationary case, $\hat{d}={\hat{d}}_{\text{diff}}+1$ else $\hat{d}={\hat{d}}_{\text{o}}$. This parameter estimate $\hat{d}$ was then converted into *α* metrics by a simple linear transform $\left(\hat{\alpha }=\left(2\hat{d}+1\right)/2\right)$.

## 4. Results

**Figure 1:**
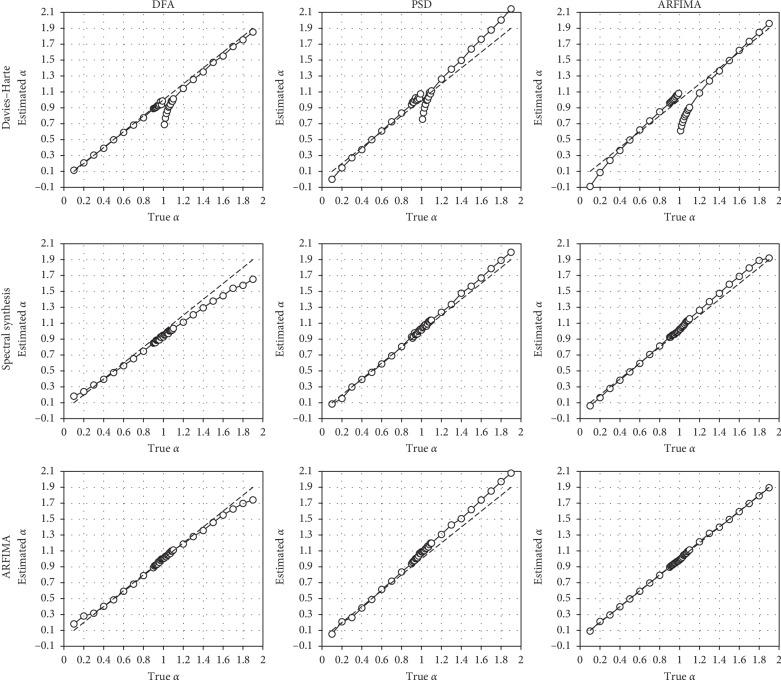
Relationship between true and mean estimated *α* values. Top row: series generated by the DH algorithm; middle row: series generated by SSM; bottom row: series generated by ARFIMA simulation. Left column: estimations performed with DFA; center column: estimations performed with PSD; right column: estimations performed with ARFIMA modeling. Dashed lines represent identity.

We present in Figure 1 the relationships between the true *α* and the mean $\hat{\alpha }$ values, for each simulation and estimation method. If we consider the global shape of results around the identity line, it seems obvious that estimation methods performed better when series were generated by the method belonging to the same domain (see the three graphs ranging along the descending diagonal). This was already observed by Eke et al. [11].

The first row depicts the results obtained by the three estimation methods with the series generated by the DH algorithm. A common bias appears in the three graphs, with a strong underestimation of *α* in fBm series, close to the 1/*f* boundary. This reveals a clear disruption in the original fGn/fBm model. This phenomenon was not observed when series were generated with the two other methods.

DFA tends to underestimate *α* for fBm series, especially when series were generated by SSM or ARFIMA modeling. Conversely, PSD tends to overestimate *α* in fBm series, especially when series were generated by the DH algorithm or by ARFIMA modeling.

**Figure 2:**
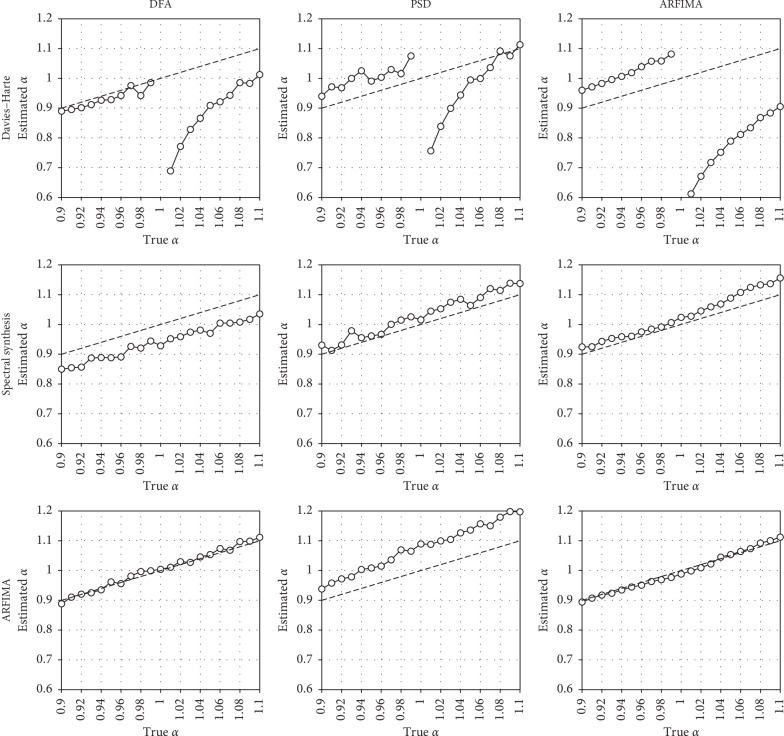
Relationship between true and mean estimated *α* values, with a focus on the 1/*f* boundary (0.9 ≤ *α* ≥ 1.1). Top row: series generated by the DH algorithm; middle row: series generated by SSM; bottom row: series generated by ARFIMA simulation. Left column: estimations performed with DFA; center column: estimations performed with PSD; right column: estimations performed with ARFIMA modeling. Dashed lines represent identity.

Figure 2 represents a focus of the previous results on the 1/*f* boundary (i.e., from *α* = 0.9 to *α* = 1.1). The first row confirms the clear disruption, around the 1/*f* boundary, for series generated with the DH algorithm. Additionally, it reveals a slight overestimation in fGn series, in the vicinity of 1/*f* noise, for PSD and ARFIMA modeling. In contrast, series generated by SSM and ARFIMA simulation present a clear continuity around the 1/*f* boundary, whatever the method of estimation. DFA tends to overestimate *α* for series generated by spectral synthesis, but not for series simulated with ARFIMA. PSD tends to overestimate *α* in this particular range. Finally, ARFIMA modeling slightly overestimates *α* for fBm series generated by SSM.

**Figure 3:**
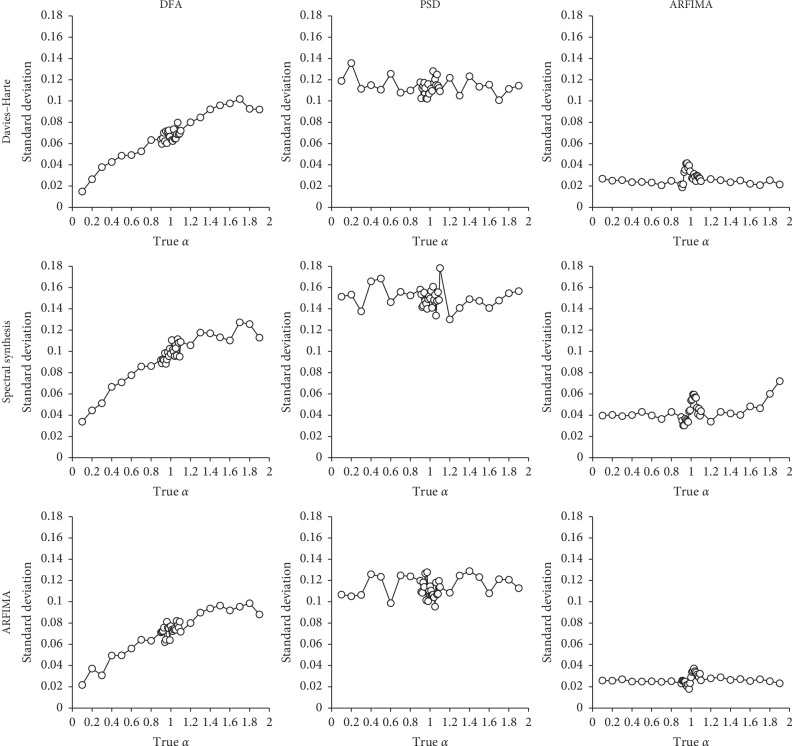
Relationship between true *α* and the variability of estimated *α* values (standard deviation). Top row: series generated by the DH algorithm; middle row: series generated by SSM; bottom row: series generated by ARFIMA simulation. Left column: estimations performed with DFA; center column: estimations performed with PSD; right column: estimations performed with ARFIMA modeling.

Finally, we present in Figure 3 the variability (standard deviation) of the $\hat{\alpha }$ samples, for each simulation and estimation method. These graphs are presented with the same scale on the vertical axe, in order to facilitate comparisons between methods. Globally, $\hat{\alpha }$ appears less variable in series generated by the DH algorithm or with ARFIMA simulation, than in those obtained from SSM, which yielded the worst results. Concerning estimation methods, DFA results show a clear increase in $\hat{\alpha }$ variability as true *α* increases. This effect is not present with PSD and ARFIMA modeling, in which variability remains stable over the whole true *α* range. PSD results reveal a high level of variability, and especially when series were generated by SSM. Conversely, ARFIMA modeling is characterized by a low variability.

## 5. Discussion

### 5.1. Davies–Harte Algorithm

The most important observation with the series generated with the DH algorithm is the strong bias for fBm series close to the 1/*f* boundary. This result, consistently obtained with the three analysis methods, should obviously be attributed to a bias in the generation technique. This result was already evidenced by Stadnitski [27], who suggested that “the observed discrepancies probably occurred due to the incapability of the Davies–Harte technique to provide fBm series with *H* close to 0. These results underline the importance of a proper data generation in simulation studies and indicate a revision of results from studies that employed the Davies–Harte technique” (pp. 144‐145).

**Figure 4:**
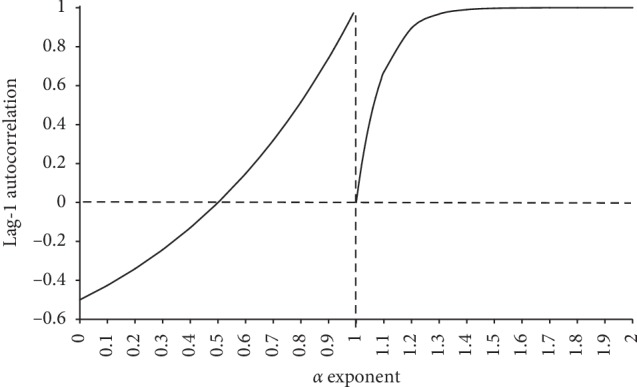
Theoretical lag-1 autocorrelation for fGn (a), based on equation (5), and for fBm (b), based on equation (33), for *α* values ranging from 0 to 2 (adapted from Delignières [28]).

Delignières [28] showed that this bias was not related to the Davies–Harte algorithm, but more fundamentally to the fGn/fBm model itself. Based on the premises of the model, and especially on the autocorrelation of fGn (equation (5)), the author derived an analytical expression for the autocorrelation of fBm:

(33) $$\begin{array}{c} \rho_{\text{fBm}}\left(\tau \right)=1-\frac{N\left(N-1\right)\tau^{2H}}{2\sum_{t=1}^{N-1}\left[\left(N-t\right)t^{2H}\right]}, \end{array}$$

where *N* is the length of the series. We report in Figure 4 the theoretical lag-1 autocorrelations, computed for fGn series ranging from *H* = 0 to 1 according to equation (5) and for fBm series ranging from *H* = 0 to 1 according to equation (33), considering *N* = 1024. These results evidence a severe breakdown of the correlation properties of series around the fGn/fBm boundary, and the limit behavior of fBm, *H* approaching 0, is uncorrelated white noise.

**Figure 5:**
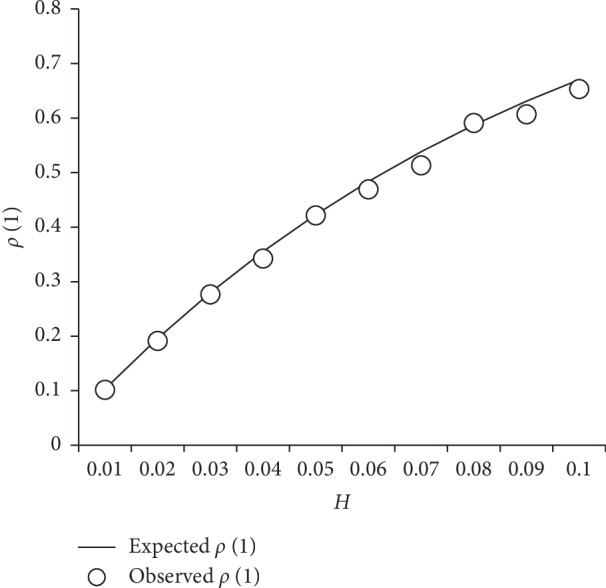
Expected and observed lag-1 autocorrelation for fBm signals. Solid line: theoretical lag-1 autocorrelation of fBm signals, for *H* ranging from 0.01 to 0.1, computed on the basis of equation (33). White circles: mean lag-1 autocorrelations observed in the series simulated with the Davies–Harte algorithm.

This suggests that fBm series, obtained by the cumulative summation of the corresponding fGn series, should in fact be fGn for very low *H* values. On the basis of equation (33), we computed the expected *ρ*_fBm_(1) values, for *H* ranging from 0.01 to 0.1, and we compared these expected values to the mean lag-1 autocorrelations observed in the series simulated with the Davies–Harte algorithm. The results are illustrated in Figure 5 and show that the correlational structure of simulated series matched closely that expected from the fGn/fBm model.

### 5.2. Spectral Synthesis Method

In contrast with the DH algorithm, SSM provides continuity around the 1/*f* boundary. This continuity was expected, as SSM works indifferently over the whole range of *β* exponents. This result could be surprising, as fGn/fBm and spectral models are often considered equivalent, representing similar properties in the time and frequency domains, respectively.

Another important result is related to the variability of estimates, which appears systematically higher in series generated by SSM than that observed with other methods of generation. This represents an important problem with this method, which seems unable to provide series sufficiently close to the expected exponents.

### 5.3. ARFIMA Simulation

ARFIMA simulation also provides a nice continuity around the 1/*f* boundary. This result is interesting, as fBm series were in that case obtained by cumulative summation of their correspondent fGn, as for the DH algorithm. Additionally, the obtained sets of series presented the lowest variability in exponent estimations, whatever the used method. These results suggest that one could get a good confidence in series generated by ARFIMA, as compared with the two other methods.

### 5.4. Detrended Fluctuation Analysis

DFA works quite well with fGn series but presents a systematic negative bias and a high level of variability for fBm series. For understanding these poor results with fBm series, as compared with other methods, it is important to keep in mind that DFA actually works on integrated series and in this case on integrated fBm. This family of overdiffusive processes is not well known, and the diffusion property exploited by DFA seems moderately appropriate with such series [9].

DFA is a very popular method, which presents the advantage to be indifferently applicable to both fGn and fBm. Some other methods gave satisfying results but are only relevant for fGn (e.g., the dispersional analysis or the rescaled range analysis [33]) or for fBm (e.g., the scaled windowed variance analysis [34]). However, researchers are often unable to know if their signals refer to any of these families, especially if these signals are close to the 1/*f* boundary. DFA allows overcoming this problem. The poor performances of DFA with fBm signals could be neglected, considering that most physiological series fall into the fGn family. In some cases, however, for example, for postural sway or gaze fluctuations, series are clearly nonstationary and should be considered as fBm. In such cases, one could consider to apply DFA on differenced series or to omit the integration step in the algorithm (see, e.g., [35, 36]).

It is worth noting that the present results were obtained with the evenly spaced version of DFA, which was proven to significantly improve the accuracy and to reduce the variability of the original method [29]. Results would have been even worse with the original algorithm.

### 5.5. Power Spectral Density

Despite the application of the refinements proposed by Eke et al. [10], PSD provided the worst estimation results. In terms of accuracy, PSD tends to overestimate *α* in fBm series, and results are particularly deceptive in terms of variability. PSD remains a quite popular method, especially because it provides appealing graphical results. The bilogarithmic representation of the power spectrum, beyond the estimation of the 1/*f* slope, can give essential indications about the presence and the nature of short-term fluctuations in the series (see, e.g., [37, 38]). However, for the specific purpose of exponent estimation, PSD seemed clearly outperformed by other methods.

### 5.6. ARFIMA Modeling

ARFIMA modeling has been neglected by most previous studies comparing fractal methods [9–11]. Rangarajan and Ding [39] developed an integrated approach of fractal analysis associating rescaled range and spectral analyses, but they never considered ARFIMA as a possible alternative or complement. However, this method provided the most accurate and less variable estimates. As indicated in the introduction, ARFIMA modeling was initially designed for accounting for stationary series. As proposed by Diebolt and Guiraud [32], we differentiated nonstationary series before the application of ARFIMA modeling, and this method gave satisfying results. We applied a very simple procedure for distinguishing stationary and nonstationary series, considering that series yielding *d* estimates equals to the upper limit obtainable with the algorithm (0.49999) should be considered nonstationary. This simple procedure provided good results, as evidenced by the nice continuity observed around the 1/*f* boundary. In the initial steps of this work, we tested the ARFIMA package [40, 41] for the matrix computing language Ox [42]. This algorithm, however, gave worse results around the 1/*f* boundary. Liu et al. [43] recently proposed a first evaluation of diverse ARFIMA programs, available on various platforms (Matlab, *R*, SAS, and OxMetrics), providing solutions for simulation, parameter estimation, and forecasting. Further investigations should be necessary for providing effective guidelines for the selection of the most relevant solutions.

In the present paper, we limited ourselves to the estimation of ARFIMA (0, *d*, 0) models. ARFIMA modeling could also account for various autoregressive and/or moving average processes that could contaminate empirical series. This method allows isolating long-range correlations in series and then to provide a better estimation of fractal exponents. Finally, a procedure using ARFIMA modeling has been proposed for gauging the effective presence of genuine long-range correlations in empirical series [26, 38, 44]. Indeed, short-term processes could sometimes mimic 1/*f*-like fluctuations, yielding false detections of long-range correlations with classical methods such as DFA [38]. The method proposed by Torre et al. [26] allows testing the relative likelihood of various ARMA and ARFIMA models and finally to conclude to the effective presence of long-range correlations.

In order to illustrate the experimental usefulness of these results, we applied the three estimation methods to a set of series of stride durations, collected during 15 min walking bouts with two groups of participants: the first one was composed of 22 young participants (28.07 yrs ± 8.88) and the second of 23 older persons (72.36 yrs ± 4.88). These data were collected during recent experiments performed in our lab [45, 46], and each series had a length of 512 data points. As indicated previously, Hausdorff et al. [47] showed that aging was characterized by a typical loss of complexity in walking dynamics, and one could expect to evidence significant differences in *α* estimates between the two groups. We present in Table 1 the results obtained with the three methods, converted in *α* metrics for comparison. In accordance with our previous results, PSD yielded a greater variability in estimates than DFA or ARFIMA. We compared the two samples with a one-way ANOVA, which did not evidence any difference between the two groups on the basis of PSD estimates. In contrast, a significant difference was found for DFA and ARFIMA estimates, with a stronger effect size for ARFIMA.

Beyond our observations about the superiority of ARFIMA modeling, in terms of accuracy and variability of exponent estimation on exact synthetic signals, this final result shows that ARFIMA modeling provides a better statistical power than DFA or PSD in the analysis of experimental data [48]. Further investigations remain necessary, however, for assessing the respective performances of these methods, especially with shorter time series, which represent an essential challenge in human behavioral experiments [9–12, 21, 29, 49].

## 6. Conclusion

This study provides a strong support for the use of ARFIMA methods, for simulation as well as for parameters estimation purposes. As previously indicated, ARFIMA methods were not considered in recent comparative studies and rarely exploited in empirical works, at least in the physiological and behavioral domains. The accuracy and the low variability of exponent estimation with ARFIMA modeling should convince researchers of the advantages of this method, especially for detecting mean differences between groups. This method should attract the attention of researchers, either for their future experiments or for revisiting their previous results.

## Figures and Tables

**Table 1:** Mean *α* estimates (standard deviation in parentheses), computed with the three methods (DFA, PSD, and ARFIMA), from series of series of stride durations, collected during 15 min walking bouts with young (*N* = 22) and older (*N* = 23) participants.

		DFA	PSD	ARFIMA
Young	Mean (*α*DFA) SD	0.878 (0.096)	1.023 (0.137)	0.887 (0.096)
Elderly	Mean (*α*DFA) SD	0.783 (0.112)	0.951 (0.196)	0.764 (0.088)
	*F* (1, 43)	9.397	1.999	20.044
	*p*	0.004	0.165	0.000
	Partial *η*^2^	0.179	0.044	0.318

## Data Availability

The time series used to support the findings of this study are available from the corresponding author upon request.

## References

[1] Gilden D., Thornton T., Mallon M. (1995). 1/*f* noise in human cognition. *Science*.

[2] Hausdorff J. M., Peng C. K., Ladin Z., Wei J. Y., Goldberger A. L. (1995). Is walking a random walk? Evidence for long-range correlations in stride interval of human gait. *Journal of Applied Physiology*.

[3] Lemoine L., Torre K., Delignières D. (2006). Testing for the presence of 1/*f* noise in continuation tapping data. *Canadian Journal of Experimental Psychology/Revue Canadienne de Psychologie Expérimentale*.

[4] Peng C.-K., Mietus J., Hausdorff J. M., Havlin S., Stanley H. E., Goldberger A. L. (1993). Long-range anticorrelations and non-Gaussian behavior of the heartbeat. *Physical Review Letters*.

[5] Hausdorff J. M. (2009). Gait dynamics in Parkinson’s disease: common and distinct behavior among stride length, gait variability, and fractal-like scaling. *Chaos: An Interdisciplinary Journal of Nonlinear Science*.

[6] Hunt N., McGrath D., Stergiou N. (2014). The influence of auditory-motor coupling on fractal dynamics in human gait. *Scientific Reports*.

[7] Kaipust J. P., McGrath D., Mukherjee M., Stergiou N. (2013). Gait variability is altered in older adults when listening to auditory stimuli with differing temporal structures. *Annals of Biomedical Engineering*.

[8] Rhea C. K., Kiefer A. W., D’Andrea S. E., Warren W. H., Aaron R. K. (2014). Entrainment to a real time fractal visual stimulus modulates fractal gait dynamics. *Human Movement Science*.

[9] Delignieres D., Ramdani S., Lemoine L., Torre K., Fortes M., Ninot G. (2006). Fractal analyses for ‘short’ time series: a re-assessment of classical methods. *Journal of Mathematical Psychology*.

[10] Eke A., Herman P., Bassingthwaighte J. B. (2000). Physiological time series: distinguishing fractal noises from motions. *Pflügers Archiv: European Journal of Physiology*.

[11] Eke A., Herman P., Kocsis L., Kozak L. R. (2002). Fractal characterization of complexity in temporal physiological signals. *Physiological Measurement*.

[12] Jennane R., Harba R., Jacquet G. (2001). Analysis methods for fractional Brownian motion: theory and comparative results. *Trait Signal*.

[13] Davies R. B., Harte D. S. (1987). Tests for Hurst effect. *Biometrika*.

[14] Fatichi S. (2009). ARFIMA simulations. https://www.mathworks.com/matlabcentral/fileexchange/25611-arfima-simulations.

[15] Saupe D., Peitgen H. O., Saupe D. (1988). Algorithms for random fractals. *The Science of Fractal Images*.

[16] Mandelbrot B. B., Van Ness J. W. (1968). Fractional Brownian motions, fractional noises and applications. *SIAM Review*.

[17] Voss R. F. 1/*f* (flicker) noise: a brief review.

[18] Granger C. W. J., Joyeux R. (1980). An introduction to long-memory time series models and fractional differencing. *Journal of Time Series Analysis*.

[19] Taqqu M. S., Teverovsky V., Willinger W. (1995). Estimators for long-range dependence: an empirical study. *Fractals*.

[20] Flandrin P. (1989). On the spectrum of fractional Brownian motions. *IEEE Transactions on Information Theory*.

[21] Delignières D., Marmelat V. (2013). Theoretical and methodological issues in serial correlation analysis. *Advances in Experimental Medicine and Biology*.

[22] Box G. E. P., Jenkins G., Reinsel G. C., Ljung G. M. (1976). *Time Series Analysis: Forecasting and Control*.

[23] Fortes M., Ninot G., Delignières D. (2005). The auto-regressive integrated moving average procedures: implications for adapted physical activity research. *Adapted Physical Activity Quarterly*.

[24] Beran J. (1994). *Statistics for Long-Memory Processes*.

[25] Beran J., Feng Y., Ghosh S., Kulik R. (2013). *Long-Memory Processes*.

[26] Torre K., Delignières D., Lemoine L. (2007). Detection of long-range dependence and estimation of fractal exponents through ARFIMA modelling. *British Journal of Mathematical and Statistical Psychology*.

[27] Stadnitski T. (2012). Some critical aspects of fractality research. *Nonlinear Dynamics, Psychology, Life Sciences*.

[28] Delignières D. (2015). Correlation properties of (discrete) fractional Gaussian noise and fractional Brownian motion. *Mathematical Problems in Engineering*.

[29] Almurad Z. M. H., Delignières D. (2016). Evenly spacing in detrended fluctuation analysis. *Physica A: Statistical Mechanics and Its Applications*.

[30] Fougere P. F. (1985). On the accuracy of spectrum analysis of red noise processes using maximum entropy and periodogram methods: simulation studies and application to geophysical data. *Journal of Geophysical Research*.

[31] Inzelt G. (2011). *Maximum Likelihood Estimators of Stationary Univariate ARFIMA (p, d, q) Processes*.

[32] Diebolt C., Guiraud V. (2005). A note on long memory time series. *Quality and Quantity*.

[33] Caccia D. C., Percival D., Cannon M. J., Raymond G., Bassingthwaighte J. B. (1997). Analyzing exact fractal time series: evaluating dispersional analysis and rescaled range methods. *Physica A: Statistical Mechanics and Its Applications*.

[34] Cannon M. J., Percival D. B., Caccia D. C., Raymond G. M., Bassingthwaighte J. B. (1997). Evaluating scaled windowed variance methods for estimating the Hurst coefficient of time series. *Physica A: Statistical Mechanics and Its Applications*.

[35] Delignières D., Torre K., Bernard P.-L. (2011). Transition from persistent to anti-persistent correlations in postural sway indicates velocity-based control. *PLoS Computational Biology*.

[36] Stephen D. G., Anastas J. (2011). Fractal fluctuations in gaze speed visual search. *Attention, Perception, and Psychophysics*.

[37] Delignières D., Lemoine L., Torre K. (2004). Time intervals production in tapping and oscillatory motion. *Human Movement Science*.

[38] Wagenmakers E.-J., Farrell S., Ratcliff R. (2004). Estimation and interpretation of 1/*fα* noise in human cognition. *Psychonomic Bulletin and Review*.

[39] Rangarajan G., Ding M. (2000). Integrated approach to the assessment of long range correlation in time series data. *Physical Review E*.

[40] Doornik J. A., Ooms M. (1999). *A Package for Estimating, Forecasting, and Simulating Arfima Models: Arfima Package 1.0 for Ox*.

[41] Ooms M., Doornik J. A. Estimation, Simulation and Forecasting for Fractional Autoregressive Integrated Moving Average Models.

[42] Doornik J. A. (2001). *Ox: An Object-Oriented Matrix Language*.

[43] Liu K., Chen Y., Zhang X. (2017). An evaluation of ARFIMA (autoregressive fractional integral moving average) programs. *Axioms*.

[44] Wagenmakers E.-J., Farrell S., Ratcliff R. (2005). Human cognition and a pile of sand: a discussion on serial correlations and self-organized criticality. *Journal of Experimental Psychology: General*.

[45] Almurad Z. M. H., Roume C., Blain H., Delignieres D. (2018). Complexity matching: restoring the complexity of locomotion in older people through arm-in-arm walking. *Frontiers in Physiology*.

[46] Almurad Z. M. H., Roume C., Delignières D. (2017). Complexity matching in side-by-side walking. *Human Movement Science*.

[47] Hausdorff J. M., Mitchell S. L., Firtion R. (1997). Altered fractal dynamics of gait: reduced stride-interval correlations with aging and Huntington’s disease. *Journal of Applied Physiology*.

[48] Kuznetsov N. A., Rhea C. K. (2017). Power considerations for the application of detrended fluctuation analysis in gait variability studies. *PLoS One*.

[49] Delignieres D., Marmelat V. (2012). Fractal fluctuations and complexity: current debates and future challenges. *Critical Reviews in Biomedical Engineering*.

